# The economic burden of mental disorders in China, 2005–2013: implications for health policy

**DOI:** 10.1186/s12888-016-0839-0

**Published:** 2016-05-11

**Authors:** Junfang Xu, Jian Wang, Anders Wimo, Chengxuan Qiu

**Affiliations:** Center for Health Economic Experiments and Public Policy, Department of Social Medicine and Administration, School of Public Health, Shandong University, Jinan, Shandong China; Aging Research Center, Department of Neurobiology, Care Sciences and Society (NVS), Karolinska Institutet-Stockholm University, Stockholm, Sweden; Division of Neurogeriatrics, Department of NVS, Karolinska Institutet, Stockholm, Sweden

**Keywords:** Costs, Economic burden, Mental disorders, Mental health, Health policy, China

## Abstract

**Background:**

Mental disorders represent a major contributor to disease burden worldwide. We sought to quantify the national economic burden of mental disorders in China.

**Methods:**

We used a prevalence-based, bottom-up approach to estimate the economic costs of mental disorders in 2005–2013 in China. Prevalence data were derived from a national survey. Cost data were derived from the electronic health records of two psychiatric hospitals that consisted of 25,289 outpatients (10 %) and inpatients (90 %) who were diagnosed with a mental disorder. Cost items included direct medical costs, direct non-medical costs, and indirect costs.

**Results:**

The total annual costs of mental disorders in China increased from $1,094.8 in 2005 to $3,665.4 in 2013 for individual patients, and from $21.0 billion to $88.8 billion for the whole society. The total costs of mental disorders in 2013 accounted for more than 15 % of the total health expenditure in China, and 1.1 % of China’s gross domestic product. If the needs of the professional care for all patients with mental illnesses were fully met, the potential economic costs would have been almost five times higher than the actual estimated costs.

**Conclusions:**

Mental disorders imposed a huge economic burden on individuals and the society in China. A nation-wide strategic action plan for preventing mental disorders and promoting mental health and well-being is in urgent need to reduce the individual and societal costs of mental illnesses.

**Electronic supplementary material:**

The online version of this article (doi:10.1186/s12888-016-0839-0) contains supplementary material, which is available to authorized users.

## Background

The World Health Organization (WHO) report (2007) estimated that worldwide 450 million people suffered from mental or behavioural disorders, placing mental disorders one of the leading causes of ill-health and disability [1]. The global burden of mental disorders increased by 37 % from 1990 to 2010 [2]. The updated WHO report in 2010 showed that ~12 % of global burden of diseases was attributable to mental disorders, which would reach ~15 % by 2020; much of the increased burden would occur in low- and middle-income countries [3]. The WHO “mental health action plan 2013–2020” highlighted the importance of promoting mental health and well-being and preventing mental disorders across the world [4].

A survey in four provinces in China (2001–2005) revealed that mental disorders affected 17.5 % of adults aged ≥18 years, which means that more than 173 million people suffered from a mental disorder in China [5]. In 2014, 4.3 million people were registered to have severe mental illnesses [6]. Chinese government has taken a series of actions in the past decade to address the mental health problems. For example, the New Healthcare Reform Plan 2009, which sought to achieve universal health care with a broad coverage of basic health insurance, incorporated major mental disorders (e.g., schizophrenia, paranoid psychosis, and bipolar affective disorders) into the public health care scheme. Furthermore, the National People’s Congress approved the first National Mental Health Law in 2012 [7], and in 2015 the central government launched the National Mental Health Working Plan 2015–2020 to promote mental health and its health care services [6]. However, owing partly to a strong social stigma attached to mental ill health, ~90 % of patients with mental disorders have never sought any professional treatment [5, 8]. Moreover, allocations of health care budget in China were heavily skewed towards somatic diseases, whereas <1 % of the total health expenditure was spent on mental disorders [3].

Data on economic burden of diseases are critical for policy-makers to set public health priorities and allocate scarce resources [9, 10]. Previous studies have included mental disorders as part of the Global Burden of Diseases [2, 3, 11], which provides valuable information to policy-makers with regard to the worldwide epidemiological burden (e.g., prevalence and incidence) and the disability-adjusted life years (DALYs) of mental disorders. In China, the economic costs of some specific types of mental disorders (e.g., depression, schizophrenia, and mood disorders) in inpatients or outpatients in different areas of China have been reported (see Additional file 1: Table S1 for a brief summary of the literature). However, the national cost data on a broad range of mental disorders are not available. Here, we seek to estimate the individual and societal economic burden attributable to mental disorders in China and discuss the potential implications for national health policy.

## Methods

### Sources of cost data

This study targeted adult patients aged ≥18 years who were diagnosed with a mental disorder. The study sample for cost estimation included inpatients and outpatients with a diagnosis of mental disorders from two psychiatric hospitals in Shandong, i.e., Shandong Center for Mental Health in Jinan (the only provincial psychiatric hospital in Shandong) for all patients in May 2005-December 2013, and Daizhuang Psychiatric Hospital in Jining, Shandong (one of the oldest psychiatric hospitals in China) for all patients in January 2012-December 2013. Mental disorders were defined according to the Diagnostic and Statistical Manual of Mental Disorders, 4th Edition and the Chinese Classification of Mental Disorders Version 3, which are consistent with the WHO International Classification of Diseases, 10th revision (ICD-10). The data were derived from the electronic health records (EHR) of the two hospitals. The EHR system documented data on demographics (e.g., age, gender, and employment), clinical diagnosis and classification (ICD-10), itemized costs (e.g., costs for drugs, examinations, and bed), and insurance information (e.g., types of health insurance, costs paid by insurance programs, and out-of-pocket costs) for all patients. We excluded patients who were diagnosed with a mental disorder due to neurological and neurodegenerative disorders (e.g., dementia), and those who were hospitalized for more than one year. In addition, because many mental disorders frequently relapse, patients might receive multiple times of care and treatment per year. Thus, we estimated the costs per patient per year rather than the costs per person time or admission. In total, the analytical sample included 25,289 patients (10 % outpatients and 90 % inpatients) who were diagnosed with a mental disorder.

### Prevalence-based bottom-up approach

We used the prevalence-based, bottom-up approach to estimate the economic costs of mental illnesses in China from both the individual and societal perspective. That is, the total economic burden was estimated based on the unit costs at individual levels, and quantified upward with the prevalence of mental disorders [12, 13]. The prevalence data of mental disorders (dementia and other neurodegenerative disorders were excluded) by age, gender, living region, and diagnosis were derived from the survey conducted in four provinces in China (2001–2005), in which the sampling frame of the survey covered 12 % of China’s adult population [5].

### Estimation of economic burden

The cost items for mental disorders included direct medical costs, direct non-medical costs, and indirect costs [14]. Direct medical costs refer to costs due to treatment and rehabilitation of mental disorders (e.g., outpatient cost, hospitalization cost, and drug cost). Direct non-medical costs mean meal expenses during hospitalization. We also accounted for costs paid by health insurance agencies for treatment and care when we estimated the economic burden from the societal perspective. Indirect costs refer to the economic loss to individual patients and the society due to the disease-related disability and premature death.

We used the direct method to estimate direct costs, and the human capital approach to calculate indirect costs following a model based on bottom-up approach (Additional file 1: Figure S1) [13, 15, 16]. Data on length of hospitalization were derived from two psychiatric hospitals in Shandong. Annual salary was estimated by accounting eight working-hours per day and 250 working-days per year. We estimated the DALYs using approaches described in the Global Burden of Disease 2010 [3]. The productivity was weighted based on demographics of China population and age-specific distribution of mental disorders [17]. Demographics, salary, gross domestic product (GDP), and mortality of mental disorders were derived from China Statistical Yearbook 2005–2013 (www.stats.gov.cn). In the base option, we assumed that 8.2 % of patients with mental disorders would seek professional treatment according to a previous study [5]. We also estimated the potential total economic burden when all patients with mental disorders would seek professional treatment. In addition, we estimated the societal economic burden by age, gender, living region, and different diagnoses of mental disorders [5]. All estimated costs were converted to US dollar (exchange rate: 1 $ ≈ 6.2 RMB, Bank of China, January 2015).

### Sensitivity analyses

Multiple sensitivity analyses were conducted to explore the potential impact of variation and uncertainty in the key input parameters on the primary estimations of economic burden. We estimated the relative economic burden by (1) using 95 % confidence interval of prevalence of mental disorders (16.6–18.5 %), instead of 17.5 % in the base option, (2) assuming that the unit health care costs increased or decreased by 10 %, and (3) considering 3.7 and 22.4 % of all patients, instead of 8.2 % [5], would seek professional care services.

## Results

Of the 25,289 patients, the mean age was 42.2 (SD 15.4) years, 49.2 % were aged 18–39 years, 50.7 % were women, and 51.0 % were rural residents. The most common mental disorders were schizophrenia, schizotypal personality disorder, and delusional disorders (36.5 %), followed by neurotic, stress-related, and somatoform disorders (15.9 %), mood [affective] disorders (15.7 %), and organic, including symptomatic, mental disorders (10.6 %) (Table 1).

**Table 1:** Characteristics of the study sample (n = 25,289)

Characteristics	No. of subjects	%
Gender, women	12,813	50.7
Age, years		
18–39	12,439	49.2
40–54	7,508	29.7
≥ 55	5,342	21.1
Marital status		
No single	15,205	60.1
Single	9,875	39.0
Missing	209	0.8
Living region		
Rural	12,896	51.0
Urban	11,717	46.3
Missing	676	2.7
Patients discharged from hospitals by years		
2005	1,025	4.1
2006	1,544	6.1
2007	1,709	6.8
2008	1,691	6.7
2009	1,556	6.2
2010	1,995	7.9
2011	2,265	9.0
2012	6,267	24.8
2013	7,237	28.6
Health insurance		
No or unavailable^a^	20,083	79.4
Yes	5,206	20.6
Types of mental disorders (ICD-10 codes)		
Organic, including symptomatic, mental disorders (F04-F09)^b^	2,678	10.6
Mental and behavioral disorders due to psychoactive substance use (F10-F19)	1,677	9.6
Schizophrenia, schizotypal personality disorder, and delusional disorders (F20-F29)	9,230	36.5
Mood [affective] disorders (F30-F39)	3,973	15.7
Neurotic, stress-related and somatoform disorders (F40-F48)	4,020	15.9
Behavioral syndromes associated with physiological disturbances and physical factors (F50-F59)	119	0.5
Disorders of adult personality and behavior (F60-F69)	1,360	5.4
Mental retardation (F70-F79)	172	0.7
Behavioral and emotional disorders with onset usually occurring in childhood and adolescence (F90-F98)	2,060	8.1

^a^Data were unavailable due to delays in implementing health insurance policy or electronic hospital information system

^b^Dementia and subtypes of dementia (F00-F03) were excluded from this category

The total annual costs per patient increased from $1,094.8 in 2005 to $3,665.4 in 2013 (Table 2). The total national annual costs for mental disorders increased from $21.0 billion in 2005 to $88.8 billion in 2013. The total costs in 2013 corresponded to 1.1 % of China’s GDP.

**Table 2:** Estimated economic burden of mental disorders by items in China, 2005–2013

Cost items	2005	2006	2007	2008	2009	2010	2011	2012	2013
Individual costs, $									
Direct medical cost	586.4	654.5	839.7	982.4	1,229.5	1,280.7	1,451.5	1,070.5	971.7
Hospitalization cost	558.9	644.8	824.4	965.3	1210.1	1264.5	1431.4	1052.4	929.4
Outpatient cost	27.5	9.7	15.2	17.1	19.4	16.2	20.0	18.1	42.3
Direct non-medical cost	28.9	35.0	39.8	52.8	50.2	33.4	50.2	26.3	25.0
Direct cost	615.3	689.5	879.5	1,035.2	1,279.7	1,314.1	1,501.7	1,096.8	996.7
Indirect cost	479.5	679.5	939.9	1,064.2	1,283.6	1,674.3	1,944.1	1,698.9	2,668.7
Total economic costs	1,094.8	1,369.0	1,819.4	2,099.4	2,563.2	2,988.3	3,445.8	2,795.7	3,665.4
Societal costs, $ in billion									
Direct medical cost	11.0	12.3	15.9	18.7	23.5	24.6	28.1	20.8	19.0
Hospitalization cost	10.5	12.1	15.6	18.4	23.1	24.3	27.7	20.4	18.2
Outpatient cost	0.5	0.2	0.3	0.3	0.4	0.3	0.4	0.4	0.8
Direct non-medical cost	0.5	0.7	0.8	1.0	1.0	0.6	1.0	0.5	0.5
Insurance cost^a^	-	-	-	-	-	5.3	7.2	5.6	15.8
Direct cost	11.5	13.0	16.7	19.7	24.5	30.6	36.3	26.9	35.3
Indirect cost	9.4	13.4	18.5	21.1	25.5	33.3	38.8	34.3	53.6
Total economic costs	21.0	26.4	35.2	40.8	50.0	63.9	75.1	61.2	88.8

^a^Data on health insurance costs were missing in 2005–2009 due to a delayed reaction to the new policy or lack of cooperation between hospitals and insurance agents

**Fig. 1:**
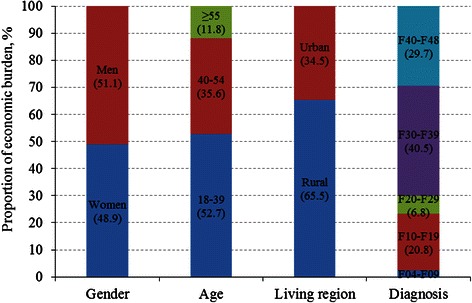
Proportion of economic burden due to mental disorders by age, gender, living region, and diagnosis

The overall costs for patients aged 18–39 accounted for 52.7 % of the total costs, 35.6 % for those aged 40–54 years, and 11.8 % for those aged 55 years and older (Fig. 1). The proportion of costs was lower for female than male patients, but higher for patients living in rural areas than those in urban regions. The overall costs for patients with mood [affective] disorders accounted for up to 40.5 % of the total costs, with the proportion being 29.7 % for neurotic, stress-related and somatoform disorders, 20.8 % for mental and behavioral disorders due to psychoactive substance use, 6.8 % for schizophrenia, schizotypal personality disorder, and delusional disorders, and 2.4 % for organic including symptomatic, mental disorders (Fig. 1).

**Fig. 2:**
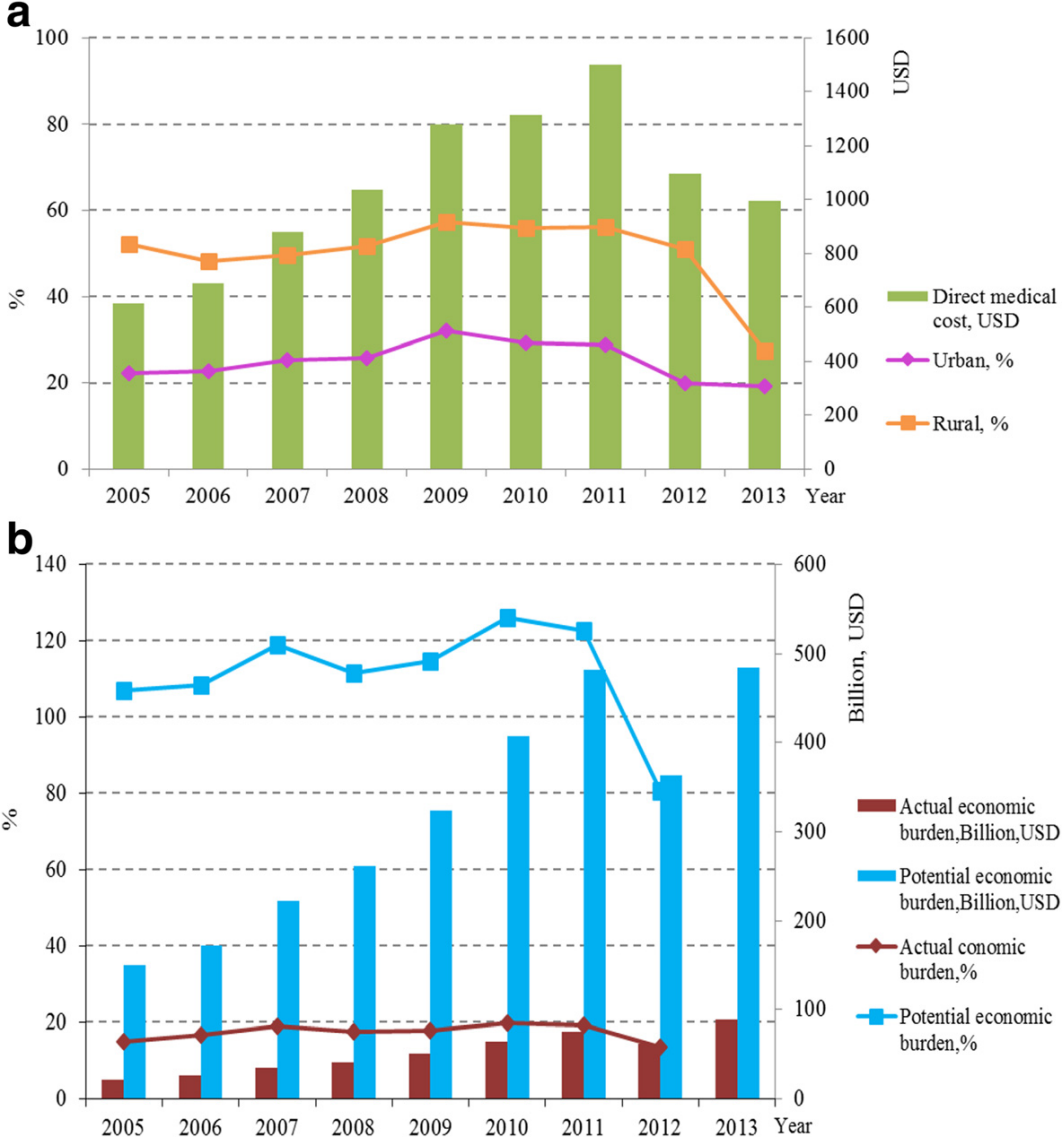
The estimated economic burden of mental disorders in China from individual (**a**) and societal (**b**) perspective, 2005–2013. **a** The estimated individual direct medical costs of mental disorders (bars, in $) and proportion of the individual direct medical costs over per capita disposable income by living region (lines, %). **b** The estimated actual societal total annual costs (bars in dark red color, in $ billion) and the potential costs when all patients would seek professional medical services (bars in blue color, in $ billion), and the corresponding proportions of actual and potential costs over total health expenditure (lines, %)

The total annual individual and national costs increased significantly in 2005–2013, with a slight decrease only in 2012 (Fig. 2). The potential economic burden would have increased from $149.6 billion in 2005 to $484.1 billion in 2013 if all patients with mental disorders had sought professional treatments.

The proportion of individual direct medical costs over per capita disposable income was relatively stable, but the economic burden of patients living in rural areas was substantially higher than that of patients in urban areas. The actual annual economic burden of mental disorders accounted for 15–20 % of the total health expenditure during 2005–2012. The proportion of potential annual economic costs over the total health expenditure was 105–130 % in 2005–2011, but decreased to 80 % in 2012 due to implementation of the national health care reform plan (Fig. 2).

**Fig. 3:**
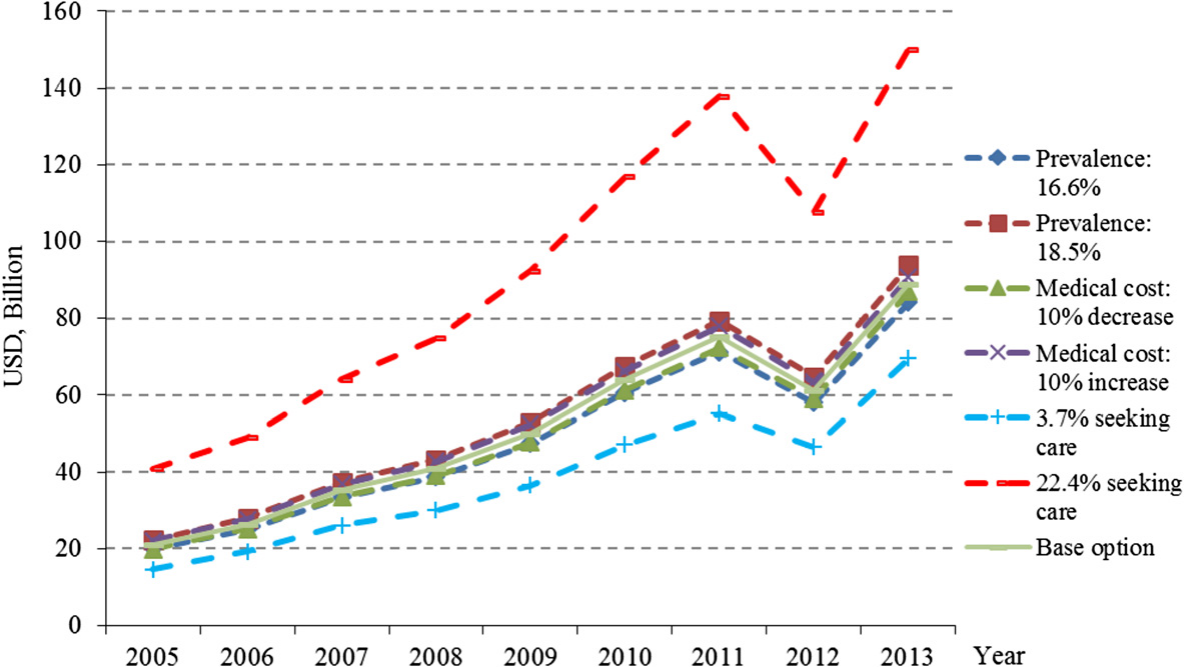
The estimated national annual economic burden of mental disorders in China, 2005–2013, according to variations in key parameters

Multiple sensitivity analyses suggested that variations in the proportion of patients who would seek professional care and treatments had substantial impact on the total economic burden, whereas variations in health care costs and prevalence of mental disorders showed relatively little impact on the total economic burden (Fig. 3).

## Discussion

### Individual and societal burden of mental disorders in China

We estimated that the total annual costs of mental disorders increased more than 3 times from 2005 ($21.0 billion) to 2013 ($88.8 billion) in China. The economic costs of mental disorders have been estimated in only a few other countries. In France, mental disorders costed ~ €109 billion in 2013, including direct, indirect costs, and intangible costs [14]. In the United States, the economic burden of major depressive disorders was estimated at $210.5 billion in 2010 [18]. The World Economic Forum estimated that the global economic burden of mental disorders exceeded those costs of any of the four major non-communicable diseases, i.e., diabetes, cardiovascular diseases, chronic respiratory diseases, and cancers [19]. However, owing to substantial variations in methodology (e.g., bottom-up vs. top-down methods and human capital vs. willingness to pay approaches) and assumption (e.g., the proportion of patients receiving and maintaining treatment) for cost estimations across studies, caution is needed when comparing findings from the literature. The economic burden due to mental illnesses in China was equivalent to ~15 % of the total health expenditure or 1.1 % of GDP in 2013. In Europe, the conservative estimate of costs of mental disorders accounted for ~3.5 % of GDP [20]. By 2030, the worldwide economic costs of mental disorders will account for nearly one-third of the projected total costs ($47 trillion) incurred by all non-communicable diseases [19].

The dramatic increase in the economic burden of mental disorders in China in 2005–2013 was driven mainly by increasing utilization of health care services (e.g., increased length of hospitalization) and increasing health care prices together with rapid economic growth. Of note, the costs of mental illnesses decreased in 2012, apparently due to the implementation of the New Healthcare Reform Plan that requests abolishment of the 15 % drug price make-up and prescription of medications following the essential medicine system for primary health care facilities [21]. However, the total costs rebounded from a sharp decline soon after. This suggests that the healthcare reform policy may not achieve the initial goal for reducing health care costs, and that failure to manage potential conflicts of interest among different sectors and stakeholders may undermine health policy [22]. Indeed, information asymmetry among medical industry, health care providers, and consumers may cause inappropriate prescriptions and overuse of medical services, which may place health care providers and pharmaceutical industry in an advantageous position to maximize profit [23].

We estimated that the direct costs of mental disorders in 2013 constituted ~40 % of the total costs. In France, the direct costs made up 20 % of the total costs while social care costs due to mental disorders accounted for 80 % [14]. The direct medical costs, which significantly increased during 2005–2013, had a greater impact on rural than urban residents. Patients in rural regions and their family faced a considerable financial burden, given that the costs accounted for ~50 % of per capita disposable income. The WHO report (2005) showed that out-of-pocket payment was the most common payment method for mental health care in low- and middle-income countries [24]. This financing model may cause households to reallocate their limited budget away from other essential needs such as foods and housing, thus leading to a catastrophic situation [25]. Furthermore, we found that indirect cost due to disability was substantial, which indicates that mental illnesses are more debilitating than certain chronic somatic diseases such as angina, arthritis, and diabetes [26]. The Global Burden of Disease Study 2010 also showed that mental disorders are the leading causes of years living with disability [27, 28].

The economic impact of mental disorders varies with patients’ characteristics. First, the individual cost was higher in male than female patients, so was the total social economic burden. This may be related to their economic security, and lower income in women may limit the utilization of mental health services. Second, the societal burden owing to disability, morbidity, and productivity loss was much higher in young (18–39 years) than middle-aged and elderly (age ≥55 years) patients. Third, although the per capita income was lower in rural than urban residents, the total social economic burden was higher in rural (65.5 %) than urban patients (34.5 %) due to larger population and higher prevalence of mental disorders in rural residents. Finally, the individual cost for patients with schizophrenia, schizotypal personality disorder, and delusional disorders was high, but the social economic burden of mood [affective] disorders was much higher due to high prevalence of these mental disorders (6.14 %) [5], which are consistent with previous reports [20].

We estimated that the potential total economic costs, if all patients with mental illnesses had sought professional care services, would have been $484.1 billion. Many mental disorders, if not properly treated and cared, may cause more severe symptoms and more fatal and non-fatal accidents to the society [29], which further increases the economic burden. Moreover, the risk of cardiovascular disease increases in patients with mental illness, but proper treatment of mental disorders may reduce the care costs of somatic disorders [30]. Thus, the unmet needs of professional care for patients with mental disorders are inexcusable.

### Strengths and limitations

This is the first study to quantify the national economic burden on a broad range of mental disorders in China. This study provides comprehensive estimates of national economic costs of mental disorders in China, which are of high relevance to policy-makers. However, our study also has limitations. First, given the considerable variations in economic development, social welfare, and health insurance systems across China, a major concern refers to the national representativeness of cost data that were derived from two psychiatric hospitals in only one province (Shandong). However, the income per capita, health expenditures, and health care facilities were generally comparable between Shandong province and the whole country. For instance, the average income per capita in Shandong province vs. China national average was $5,374 vs. $5,893, $276 vs. $291 for health expenditure per capita, $1,080 vs. $1,125 for hospital costs per capita, and 1.86 vs. 1.79 for psychiatric beds per 100,000 population (data sources: China Statistical Yearbook at www.stats.gov.cn and China Health Statistics Yearbook at www.nhfpc.gov.cn). Second, the total economic burden due to mental disorders might have been underestimated because we were not able to account for certain direct and indirect costs of mental disorders due to lack of reliable data, such as costs of informal care and social care (e.g., day care and patient follow-ups) for severe mental illness, costs of accidental and non-accidental injuries (e.g., suicide and traffic accidents) attributable to mental disorders, and other direct non-medical costs (e.g., transportation costs) of patients and accompanying family members when seeking treatment and care. On the other hand, the bottom-up approach might lead to overestimates of the disease costs when applying the costs of individual patients from psychiatric hospitals to those patients from the general hospitals or from population surveys, although we accounted for the proportion of patients seeking care in our estimations [31]. Third, the use of length of stay may overestimate the economic costs of unemployed patients, and human capital approach is subject to market imperfections, although we gave different weights of productivity to unemployed people (e.g., retirees). Finally, although we did account for the changes over time in the proportion of people’s living region (urban vs. rural areas), income (per capita wage), and economic development (GDP) from 2005 to 2013, the impacts of factors such as population aging, changes in health care and insurance policy, and improvement in medical technology and treatments could not be fully addressed.

### Implications for national strategic plan and health policies

Maintaining mental health and well-being has a profound impact on an individual’s life and the whole society [32]. Thus, a comprehensive and coordinated response for mental health requires partnership with multiple public (e.g., health, education, employment, and justice) and private (e.g., charity and non-profit organizations) sectors. The current mental health system in China relies heavily on hospital inpatient care, which leads to a high economic burden for patients and society. Thus, the provision of accessible, dependable, and affordable mental health care should be a priority [33]. This may be achievable by integrating mental health care reform with the ongoing primary health care reform in China. In addition, the development of mental health information system and standardized data collection of health care costs, and rigorous economic evaluation are important to inform the policy-makers [34]. Furthermore, although the current basic medical insurance programmes partly covered costs of mental disorders, the proportion of reimbursement for mental health care costs remained low. Policy-makers are therefore advised to increase the reimbursement rates for inpatient and outpatient care costs. Finally, government may encourage non-governmental organizations and private sectors to share responsibilities with governments in improving mental health care and information surveillance by adopting preferential policies (e.g., tax deduction, exemption, or financial incentives).

## Conclusions

This study suggests that mental disorders in China have posed substantial economic burden at individual and societal levels. The potential economic impact, when the needs of health care services for all patients with mental illness are fulfilled, would be much greater. The government should give mental health a greater priority by developing strategic action plans to promote mental health and well-being and to improve mental health care system. The economic burden of mental disorders is likely to be reduced by scaling up mental health care services, and by assuring early intervention to prevent mental disorders and avoid progression from mild mental disorders to severe illnesses and disability.

### Ethics approval and consent to participate

This study was approved by the Medical Ethics Committee at Shandong University School of Medicine in Jinan, Shandong, China. There was no need of informed consent because the study only involved retrospective reviews of the electronic medical records of patients, and patients’ identity was protected.

### Consent for publication

Not applicable.

### Availability of data and materials

By contact with the corresponding author Prof Jian Wang.

## Additional file

Additional file 1: Figure S1. The economic models used in the estimation of economic burden of diseases. Table S1 A summary of studies on economic costs of mental disorders in China. (DOC 95 kb)

## Abbreviations

DALYs: disability-adjusted life years
EHR: electronic health record
GDP: gross domestic product
SD: standard deviation
WHO: World Health Organization
