# Supplementary material for: The economic burden of mental disorders in China, 2005–2013: implications for health policy
Source: BMC Psychiatry. 2016 May 11;16:137. doi: 10.1186/s12888-016-0839-0 (PMC4864926; doi:10.1186/s12888-016-0839-0)
Supplement: Additional file 1: Figure S1. — The economic models used in the estimation of economic burden of diseases. Table S1 A summary of studies on economic costs of mental disorders in China. (DOC 95 kb) [file 12888_2016_839_MOESM1_ESM.doc]

(

)

**Direct economic burden:**

=

**+**

**Direct economic burden**

Direct medical cost

Society: direct costs

Direct non-medical cost

Individual: direct costs

Number of patients

+

=

Individual: direct costs

Insurance cost per patient

*

*

(

DALYs

GDP per person

*

) *

+

=

Discount rate

Individual loss of productivity

Number of patients

Societal indirect cost

Productivity weight

LOS due to illness

Daily salary

Individual loss of productivity

*

=

*

**Indirect economic burden:**

**Figure S1** The economic models used in the estimation of economic burden of diseases.

DALYs = disability-adjusted life years, GDP = gross domestic product, LOS = length of stay.

**Table S1 A summary of studies on economic costs of mental disorders in China**

| **Study** | **Subjects or mental disorders** | **Sample size** | **Perspective** | **Data sources** | **Estimated costs (RMB)** |
| --- | --- | --- | --- | --- | --- |
| Yang, et al. [1] | Inpatients (mental disorders) | 7,684 | Individuals | HIS | 10,005 per person |
| Chen, et al. [2] | Outpatients (depression) | 652 | Individuals | Questionnaire survey | 6,000-7,000 per person |
| Huang, et al. [3] | Mood disorders | 226 | Individuals | Questionnaire survey | 70,387.4 per person |
| Xu, et al. [4] | Elderly inpatients | 1,868 | Individuals | HIS | 5,000 per person |
| Xu, et al. [5] | Military patients | - | Individuals | HIS | 160-600 per day |
| Ye, et al. [6] | Outpatients (mental disorders) | 2,000 | Society | Questionnaire survey | 5,912 per person |
| Xing, et al. [7] | Inpatients (mental disorders) | 3,405 | Individuals | HIS | 4,000-6,000 per person |
| Yang, et al. [8] | Schizophrenia | - | Society | EHR | Direct costs: 2-3 million,  indirect costs: 60-90 million |
| Huang, et al. [9] | Schizophrenia | 40,000-90,000 person-times | Society | Health insurance database | Direct costs: 9-20 million,  indirect costs:1000 million |
| Guo, et al. [10] | Schizophrenia | 316 | Society | HIS | Direct costs: ~400,000,  indirect costs: ~60 million |
| Xu, et al. [11] | Inpatients (mental disorders) | 15,721 | Individuals | EHR | 11,949 per person |
| Liang, et al. [12] | Inpatients (mental disorders) | 1,732 | Individuals | HIS | 5,000-6,000 per person |
| Hu, et al. [13] | Depression | 505 | Society | Questionnaire survey | 51,370 million |

Abbreviations: EHR, electronic health records; HIS, hospital information system.

**References for supplemental Table 1**

1. Yang SL, Qian MC, Lu W, Wang CS, Chen HZ, Fei JF, et al. Cost of treating medical conditions in psychiatric inpatients in Zhejiang, China. Shanghai Arch Psychiatry. 2011;23:329-36.

2. Chen XB, Ji JL, Zhou XD, Chen HY, Sheng F. The economic burden of outpatients with depression in Shanghai. The Second Annual Conference of Preventive Medicine of Shanghai. 2006;9:259-62.

3. Huang HH, He YL, Chen W, Zuo S, Wang Y, Zhou ZY. Study on economic burden of mood disorder inpatients from one Tertiary Mental Health Hospital in Shanghai. Chin Health Econ. 2011;30:29-31 (in Chinese).

4. Xu JF, Yu FH, Wang J. The hospital costs of the elderly with mental disorders and path analysis of influencing factors. Chin J Health Statistic. 2014;31:475-7 (in Chinese).

5. Xu J, Sun Q, Wang CL. Analysis of influence factors of bed cost daily on military mental healthcare and countermeasures. Military Med J Southeast China. 2014;16:511-3 (in Chinese).

6. Ye CW, Huang ML, Hu JB, Xu WJ, Xu Y. The outpatient cost and influencing factors of mental disorders. Annual Conference of Psychiatry in Zhejiang Province. 2007;10:212-4.

7. Xing JS, Yu FH, Li RJ. Analysis on hospitalization expenses of the 3405 mental patients. Guide of China Medicine. 2010;8:172-4 (in Chinese).

8. Du LZ, Sun LH. The economic burden of schizophrenia. Chin J Prev Contr Chron Dis. 2013;21:621-3 (in Chinese).

9. Huang Y, Liu GE, Liu YH, Wang CZ, Ren XX, Zhang H. Economic burden of schizophrenia: based on medical insurance database from Guangzhou. Chin Health Econ. 2014;33:62-5 (in Chinese).

10. Guo J, Bao HL, Yin SF. Research of the disease burden of schizophrenia in Xuancheng, Anhui Province. Chin General Practice. 2015;18:3704-7 (in Chinese).

11. Xu JF, Wang J, Liu R, Xing JS, Su L, Yu FH, et al. Mental health inpatient treatment expenditure trends in China, 2005-2012: evidence from Shandong. J Ment Health Policy Econ. 2014;17:173-82.

12. Liang JQ, Yu M. The hospital costs of 1732 patients with mental disorders. Med J Chin People’s Health. 2014;26:89-92 (in Chinese).

13. Hu TW, He Y, Zhang M, Chen N. Economic costs of depression in China. Soc Psychiatry Psychiatr Epidemiol. 2007;2:110-6.
